# Spectrophotometric Estimation of Olmesartan Medoxomil and Hydrochlorothiazide in Tablet

**DOI:** 10.4103/0250-474X.62245

**Published:** 2010

**Authors:** A. R. Rote, P. D. Bari

**Affiliations:** Department of Pharmaceutical Chemistry, MGV's Pharmacy College, Panchavati, Mumbai Agra Road, Nashik-422 003, India

**Keywords:** Hydrochlorothiazide, Olmesartan medoxomil, UV spectrophotometry

## Abstract

A simultaneous determination of olmesartan medoxomil and hydrochlorothiazide by absorption ratio spectrophotometric method has been developed in combined tablet dosage form. The method is based on measurements of absorbance at isoabsoptive point. The Beer's law obeys in the range of 10–30 μg/ml for both olmesartan medoxomil and hydrochlorothiazide respectively. The proposed method was validated by performing recovery study and statistically.

Olmesartan medoxomil (OLM) is a prodrug and is hydrolyzed to the active olmesartan during absorption from the gastrointestinal tract. Olmesartan is a selective AT1 subtype angiotensin II receptor antagonist. It is chemically, 2,3-dihydroxy-2-butenyl4-(1-hydroxy-1-methylethyl)-2-propyl-1-[p-(o-1Htetrazol-5-ylphenyl)benzyl]imidazole-5-carboxylate, cyclic-2,3-carbonate. Hydrochlorothiazide (HCT) is one of the oldest and widely used thiazide diuretics. A literature survey revealed that OLM is not yet official in any pharmacopoeia. Several analytical methods have been reported for the determination of olmesartan medoxomil in biological fluids includes LC-MS-MS[1], degradation product HPLC[2], HPTLC[3]. One analytical method was developed for determination of HCT and OLM in combination HPLC[4], The USP described an HPLC method for determination of HCT from tablet formulation[5].

A double beam, Shimadzu 2450 UV/Vis spectrophotometer connected to a HCL computer loaded with UV Probe 2.21 Software was used in the current investigation. OLM and HCT were supplied by Glenmark Pharmaceutical Ltd and Ajanta Pharmaceuticals Ltd, respectively. Sodium hydroxide and double distilled water were used for this work. A commercial pharmaceutical preparation Olmesar-H of Macleod, Gujarat, India (containing OLM 20 mg and HCT 12.5 mg/tablet) was used for analysis.

Standard stock solution 1.0 mg/ml each of OLM and HCT were prepared in 25 ml 0.1 N NaOH. A further dilution of stock solution was made with 0.1 N NaOH to get a working standard solution of 100 μg/ml of both drugs. Then 10 ml of OLM and 6.25 ml of HCT standard stock solution of each drug was taken in 50 ml volumetric flask and diluted with 0.1 N NaOH up to the mark to get 200 μg/ml of OLM and 125 μg/ml of HCT. This solution used as standard working mixture solution.

From the standard working mixture solution of OLM and HCT, 1 ml of working mixture were taken in 10 ml volumetric flask containing 20 μg/ml of OLM and 12.5 μg/ml of HCT were prepared using 0.1 N NaOH. The standard mixtures prepared were then scanned over the range of UV 200 to 360 nm. The spectra were then obtained and absorbance was measured at selected wavelengths 260.0 nm (isoabsorptive point) and 272.8 nm for both the drugs. The concentrations of OLM and HCT were calculated by inserting the absorbance values in the Eqns.

(1) $$\mathrm{Cx}=(\mathrm{Qm}-\mathrm{qy})/(\mathrm{Qx}-\mathrm{Qy}){\mathrm{xA}}_{1}/{\mathrm{ax}}_{1}$$

(2) $$C_{Y}=(\mathrm{Qm}-\mathrm{qx})/(\mathrm{Qy}-\mathrm{Qx}){\mathrm{xA}}_{1}/{\mathrm{ay}}_{1}$$,

where Cx and C_Y_ are concentration of OLM and HCT (g/100 ml), respectively, A_1_ and A_2_ are absorbance of mixture at 248.6 nm and 272.8 nm, respectively, ax_1_ and ax_2_ denote absorptivity of OLM at 260.0 nm and 272.8 nm, respectively, ay_1_ and ay_2_ represent absorptivity of HCT at 260.0 nm and 272.8 nm, respectively, Qx and Q_Y_ are ratios of absorptivity of OLM and HCT at 272.8 nm and 260.0 nm, respectively and Qm is the ratio of absorbance of the mixture at 272.6 nm and 260.0 nm.

Linearity was constructed in the range 10-30 μg/ml for both OLM and HCT, respectively. This shows regression coefficient 0.9999 for OLM at 248.6 nm and 0.9991 for HCT at 272.8 nm. The statistical parameters were calculated. Twenty tablets were weighed accurately and powdered. Powder equivalent to 20 mg of OLM (containing 12.5 mg of HCT) was weighed and transferred to 100 ml volumetric flask and dissolved in 25 ml methanol by shaking the flask for 15 min and volume was made up to the mark with 0.1 N NaOH. The solution was filtered through Whatman filter paper No. 41. One millilitre aliquot of sample stock solution was transferred to 10 ml volumetric flask and volume was made up to mark with 0.1 N NaOH to get concentration of 20 μg/ml of OLM and 12.5 μg/ml of HCT. The solutions were then scanned over the range of 200 to 360 nm. The spectra were obtained and absorbance was measured at selected wavelengths 260.0 nm (isoabsorptive point) and 272.8 nm for both the drugs. The concentrations of OLM and HCT were calculated by putting the absorbance values in Eqns. 1 and 2.

The accuracy of the proposed method was checked by performing recovery study by addition of standard drug solution to preanalysed tablet sample solution at three different concentration levels (80, 100 and 120%) within the range of linearity for both the drugs. The wavelengths selected for this method should be wavelength (λ_max_) one of the component and second one was the isoabsorptive point where the absorptivity of both components was same which is shown in fig. 1. OLM shows maximum absorption at wavelength (λ_max_) 248.6 nm, where as the HCT shows maximum absorption at wavelength (λ_max_) 272.8 nm and 260.0 nm is the isoabsorptive point of both drugs. The analytical data for the linearity range shows correlation coefficients of 0.9999 for Olmesartan medoxomil and 0.9991 for HCT at 248.6 nm and 272.8 nm, respectively which is shown in Table 1. For quantitative analysis, concentration of OLM and HCT in tablet sample were determined by using Eqns. 1 and 2, results are shown in Table 2. The detection limits were 0.41 μg/ml for OLM and 0.44 μg/ml for HCT, while quantification limit were 1.25 μg/ml for OLM and 1.33 μg/ml for HCT. The proposed method was validated by performing recovery study. Recovery was in the range of 99.97-100.62 % for OLM and HCT respectively, the standard deviation was found to be less than 2%, shows the high precision of the proposed method The method can be used for the routine quality control analysis of the OLM and HCT in combined dosage form.

**Fig. 1:**
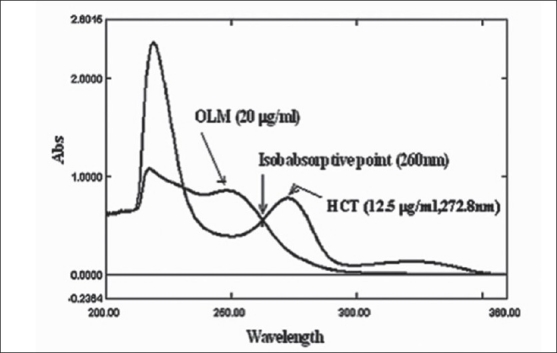
Overlay of Spectra of OLM and HCT OLM is olmesartan medoximil and HCT is hydrochlorthiazide

**TABLE 1:** OPTICAL CHARACTERISTICS OF PROPOSED METHOD

Parameter	OLM	HCT
Beer' law limit (μg/ml)	10-30	10-30
Régression Equation (y = mx + c)	-	-
Slope (m)	0.043	0.0526
Intercept (c)	-0.0031	-0.0103
Correlation coefficient	0.9999	0.9991
Limit of detection (LOD, μg/ml)	0.41	0.44
Limit of quantitation (LOQ, μg/ml)	1.25	1.33

**TABLE 2:** RESULTS OF COMMERCIAL TABLET FORMULATION ANALYSIS

Parameter	OLM	HCT
Label claim(mg/tablet)	20	12.5
Mean*	100.18	100.75
±Standard Deviation	0.5007	0.4256
±SE	0.2239	0.1903
% RSD	0.4998	0.4224

* average of 5 determinants
